# Data on cell survival, apoptosis, ceramide metabolism and oxidative stress in A-494 renal cell carcinoma cell line treated with hesperetin and hesperetin-7-O-acetate

**DOI:** 10.1016/j.dib.2018.08.065

**Published:** 2018-08-29

**Authors:** Mahdi Mashhadi Akbar Boojar, Masoud Mashhadi Akbar Boojar, Sepide Golmohammad, Iraj Bahrehbar

**Affiliations:** **a** Department of Pharmacology and Toxicology, Baqiyatallah University of Medical Sciences, Tehran, Iran; b Experimental Medicine Research Center, Tehran University of Medical Sciences, Tehran, Iran; c Department of Cell and Molecular Biology, Faculty of Biological Sciences, Kharazmi University, Tehran, Iran

**Keywords:** Hesperetin, Ceramide, Ceramidase, Sphingomyelinase, Superoxide dismutase

## Abstract

Ceramide pathway is a key regulator in cell proliferation and apoptosis and oxidative stress up-regulate ceramides. Acid ceramidase (ACDase), neutral sphingomyelinase (NSMase) and glucosylceramide synthase (GCS) are critical enzymatic systems in ceramide metabolism. Our data represent the comparative assessment of Hesperetin (Hst) and hesperetin-7-O-acetate (HTA) effects on A-494 renal carcinoma cells include cell survival, caspase-3 and 9 activities, total cellular ceramide and the activities of ACDase, NSMase, GCS and superoxide dismutase (SOD). Data reveals potentiating effects of both HTA and Hst on ceramide pathway and may offer a novel tool in human renal cell carcinoma therapy.

**Specifications Table**

**Table:** 

Subject area	Biology
More specific subject area	Apoptosis, ceramide, oxidative stress
Type of data	Graphs
How data was acquired	Fluorescence and spectrophotometry analysis
Data format	Analyzed
Experimental factors	A-494 cells were treated with hesperetin or hesperetin-7-O-acetate
Experimental features	Cells were exposed to different concentrations of hesperetin or hesperetin-7-O-acetate and after 48 h, they were prepared for spectrofluorometric assessment of related substance.
Data source location	Baqiyatallah University of Medical Sciences, Tehran, Tehran Province, Iran
Data accessibility	The data are available with this article.

**Value of the data**

• Data including ceramide up-regulation and ceramide-mediated apoptosis after treatment with hesperetin derivatives may be of value to the researchers working in renal cell carcinoma and other related cancer cell lines.
• Data showing that 7-O-acetylation of hesperetin increases the cytotoxic activity against A-494 tumor cells may be of potential value for the scientists working in the field of flavonoids.
• Data showing that hesperetin effects are mediated by regulating the ceramide metabolizing enzymes and oxidative stress may be useful for the researchers working in the cell signaling pathways.

## 1 Data

Acid ceramidase (ACDase), neutral sphingomyelinase (NSMase) and glucosylceramide synthase (GCS) are critical enzymatic systems in ceramide metabolism [1], [2], [3]. Present data is about Hesperetin (Hst) and Hesperetin-7-O-acetate (HTA) effects on A-494 renal carcinoma cells. Cell survival, caspase-3 and 9 activities, total cellular ceramide and the activities of ACDase, NSMase,GCS, and superoxide dismutase (SOD) determined with fluorescence and spectrophotometry analysis in examined cells which treated with different concentrations of Hst and HTA (0–200 μM).

**Fig. 1:**
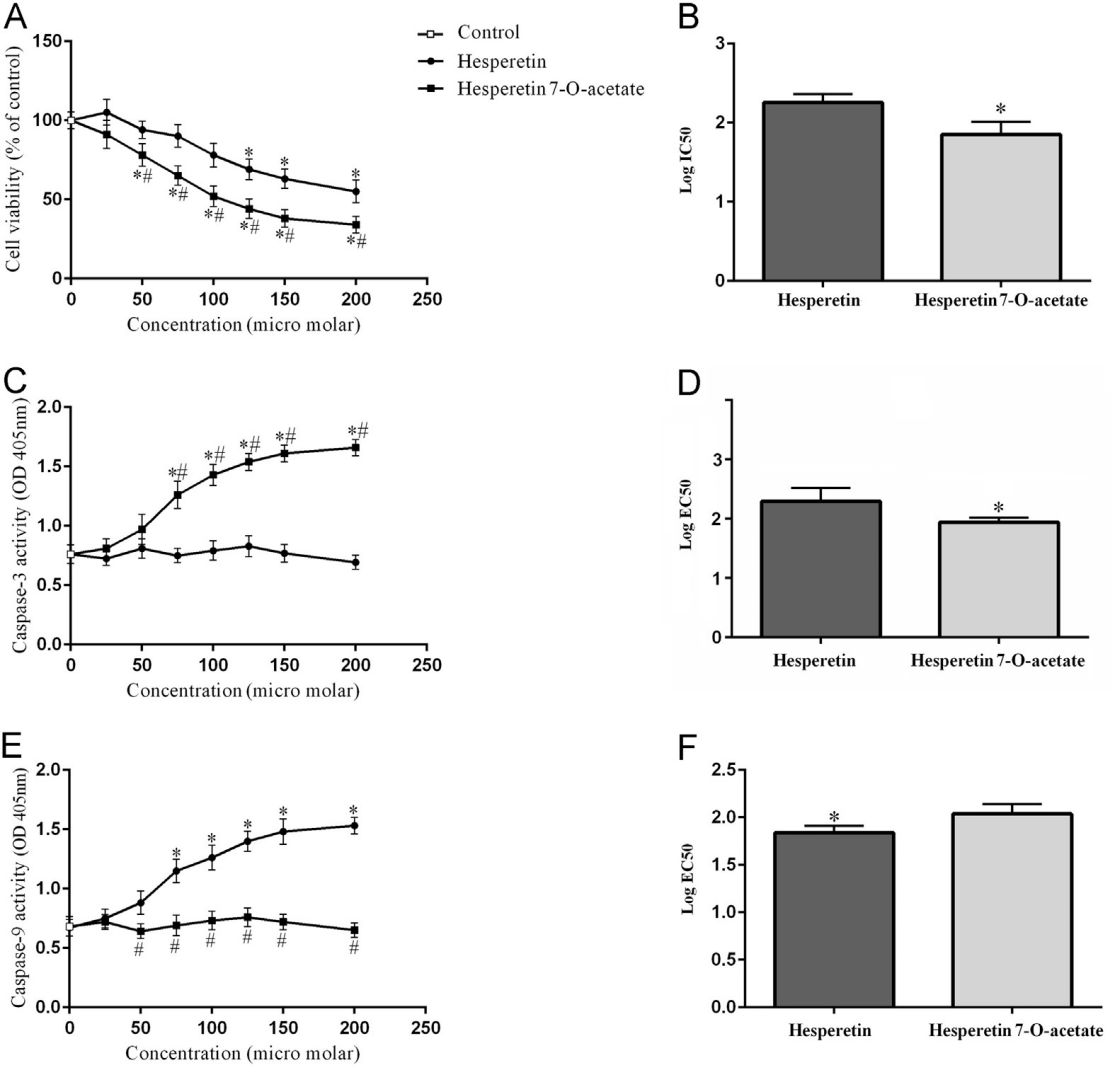
Cell survival and activities of caspases 3 and 9 in A-494 renal carcinoma cells after 48 h exposure to flavonoids. The biological response of each group (*n* = 3) in different concentrations was evaluated separately and the results were shown as mean ± S.D. A, C and E: Cell survival percentage and activities of caspases 3 and 9 after exposure to flavonoids were evaluated by MTT assay and optical absorption measurements of p-nitroanilide. * Significant difference compared to the control group (normal saline). # Significant difference of Hst compared to HTA at the same concentration (*P* < 0.05). B, D, and F: Comparison of IC50 and EC50 for Hst and HTA to evaluating cell survival and caspase activity of 3 and 9. * Significant differences in Hst compared to HTA.

Both Hst and HTA reduced cell survival and in this regard, HTA was more potent than Hst. Caspase 3 activity with HTA and caspase 9 with Hst increased significantly in a dose-dependent fashion (Fig. 1). Caspase 9 is involved in intrinsic and caspase 3 in the common pathway of apoptosis [4].

**Fig. 2:**
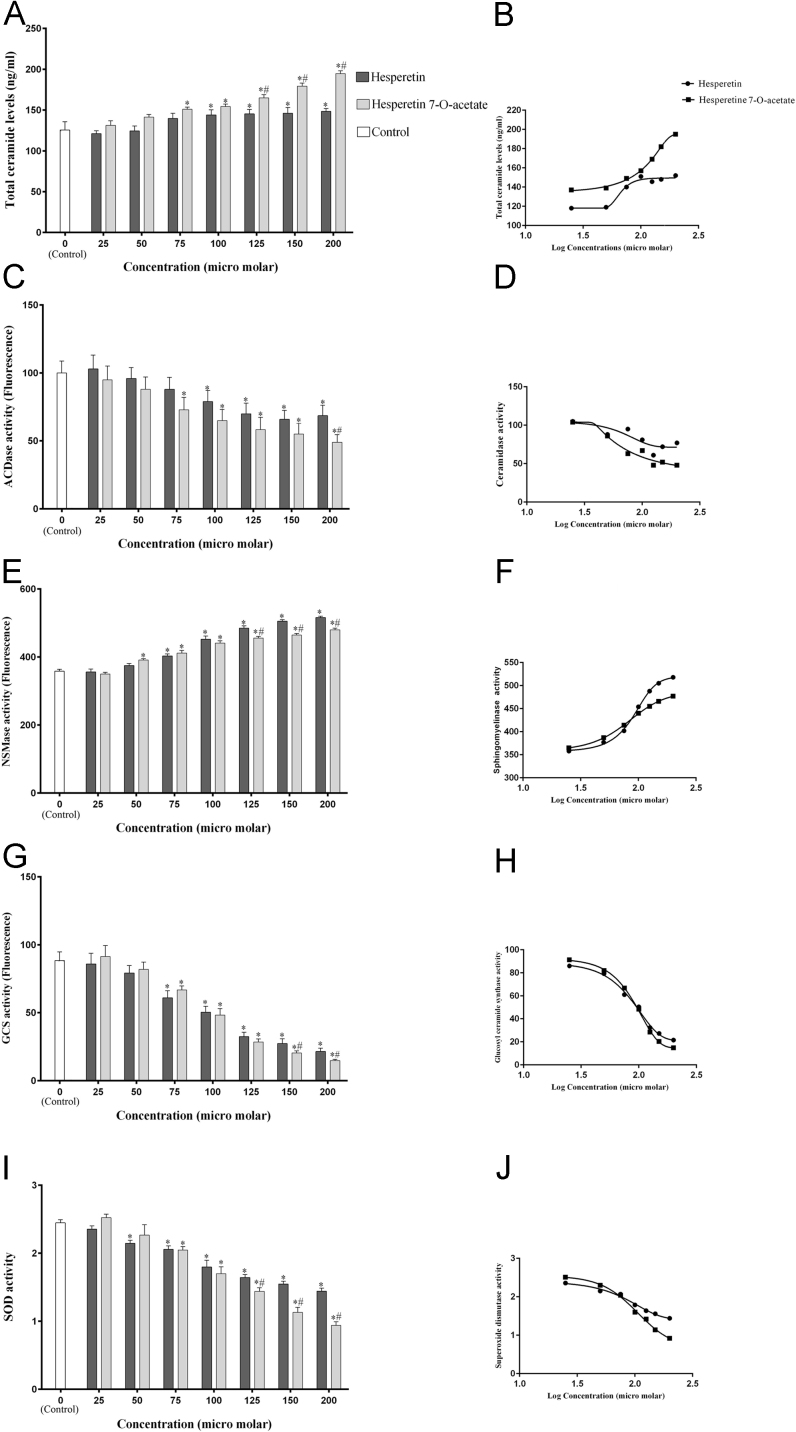
Evaluation of total levels of ceramide and the activities of enzymes involved in ceramide metabolism and oxidative stress in A-494 renal carcinoma cells after 48 h exposure to flavonoids. The biological response of each group (*n* = 3) in different concentrations was evaluated separately and the results were shown as mean ± S.D. A, C, E, G and I: Total ceramide levels and activities of ACDase, NSMase, GCS and SOD enzymes after exposure to the flavonoids were evaluated by using the fluorescence or UV/visible spectrophotometer to measure light absorbance at 252/483, 400/550, 595, 470/530 and 560 nm respectively. *Significant difference compared to the control group. #Significant difference of Hst compared to HTA at the same concentration. B, D, F, H, and J: Logarithmic curve of concentration - biological response for each test.

According to these data, total cellular ceramide increased somewhat at higher concentrations of Hst and HTA. The activity of ceramide accumulator enzyme (NSMase) increased and the activity of ceramide depletory enzymes (ACDase, GCS) attenuated efficiently by investigated compounds. The higher concentration of Hst was more effective than HTA for SOD suppression (Fig. 2).

Both Hst and HTA had cytotoxic effects in A-494 renal carcinoma cells that were originated from ceramide up-regulation. Data may suggest a new molecular mechanism of hesperidin derivatives and offer a novel tool in renal cell carcinoma.

## 2 Experimental design, materials, and methods

### 2.1 Reagents, cell line, and cell cultures

The human renal carcinoma cell line A-494 was prepared from Institute Pasteur Medical Center. They were placed in Dulbecco׳s modified Eagle׳s medium supplemented with fetal bovine serum (FBS), L-glutamine, HEPES Na and penicillin-streptomycin antibiotics. The cells were cultured in a humidified incubator at 37 °C with 5% of CO_2_. Hesperidin, hesperetin-7-O-acetate, ceramide and other reagents were bought from Sigma Chemical Co., St. Louis, MO, USA.

In each experiment, the cells were suspended at a concentration of 1 × 10^6^ cells/mL and treated separately with increasing concentrations of hesperidin and hesperetin-7-O-acetate (0, 25, 50, 75, 100, 125, 150 and 200 μM) and incubated for 48 h. The tests were repeated three times independently.

### 2.2 Cell viability

The 3-(4,5-dimethylthiazol-2-yl)-2,5-diphenyl tetrazolium bromide (MTT) colorimetric assay was performed for assessment of cell viability according to F.M. Young and J.M. Posimo instructions [5], [6]. Briefly, after separate incubation of the A-494 cells with Hst and HTA, cells were washed and then treated with MTT solution to reach the final concentration of 0.5 mg/mL and after 3 h, dimethyl sulfoxide added. The optical density of Formazan was determined by using spectrophotometer at 540 nm and the reference wavelength at 630 nm. The results were presented as survival percentages compared to controls.

### 2.3 Caspases activities

The assessment of caspase-3 and 9 activities are based on the hydrolysis of the peptide substrate by caspases-3 and 9, resulting in the generation of p-nitroaniline (pNA) as a chromophore agent. In summary, caspase assay buffer (4-(2-hydroxyethyl)-1-piperazine ethane sulfonic acid (HEPES) [pH 7.5], 3-[(3-cholamidopropyl) dimethyl lammonio]-1-propane sulfonate (CHAPS), phenylmethanesulfonyl fluoride (PMSF), dithiothreitol (DTT) and ethylenediaminetetraacetic Acid (EDTA)) was added to the cell suspension and after centrifugation, 50 μg of protein from the supernatants were added to each caspase substrates. Asp-Glu-Val-Asp-pNA and N-acetyl-Leu-Glu-His-Asp-pNA were the substrates for caspase-3 and 9 respectively. After the incubation period, the absorbance of pNA was determined at 405 nm [7].

### 2.4 Quantifying of total ceramide

Ceramide molecules in samples hydrolyzed to sphingosine with the recombinant human acid ceramidase. Then according to the instructions used by H. Xingxuan, Naphthalene-2,3-dialdehyde (NDA) as the fluorogenic label was added and after 25 min, sphingosine-NDA separated by reverse-phase high-performance liquid chromatography. The fluorescent derivatives were determined at the excitation/emission wavelength of 252 and 483 nm [8].

### 2.5 ACDase activity assays

After 24 h incubation of cells with NBD-ceramide (as a fluorogenic substrate) in sodium acetate buffer and Triton X-100, the reaction was stopped by adding methanol-chloroform. Then the supernatant was collected and this tube (as un-cleaved substrate) was evaluated in a fluorescence spectrophotometer (in the UV range 400 excitation/550 nm emission). At the same time, another test tube such as above was prepared and the cell extracts were added to it. Then Fluorescence yields determined in the UV range 430 excitation/550 nm emission. Absorption ratio of cleaved/un-cleaved NBD-ceramide was considered as an ACDase activity indicator as described above [9].

### 2.6 NSMase activity assays

The activity of this enzyme initiates by adding the substrate of sphingomyelin to the cellular extracts: sphingomyelin is converted to ceramide and phosphorylcholine. Samples were kept on ice for 15 min and centrifuged at 14,000*g* for 20 min at 4 °C. 100 μl of each supernatant were incubated for 1 h at 37 °C with working solution. After choline generation, it is used to provide hydrogen peroxide in a reaction catalyzed by choline oxidase. Finally, with peroxidase as a catalyst, hydrogen peroxide reacts with sodium N-ethyl-N-(2-hydroxy-3-sulfopropyl)-3,5-dimethoxylaniline (DAOS) and 4-aminoantipyrine to generate Resorufin with an optimal absorption at 595 nm [10].

### 2.7 GCS activity assays

To valuation of GCS activity, the fluorescent substrate C6-4-nitrobenzo-2-oxa-1,3-diazole (NBD)-ceramide and a normal phase HPLC were used. Acceptor substrate, C6-NBD-Ceramide and, lecithin were mixed in 100 μl of ethanol and then the solvent was evaporated. After adding water the obtained mixture was sonicated to form liposomes. For the GCS assay, the reaction mixture contains UDP-Glc, EDTA, C6-NBD-Ceramide liposome and the appropriate amount of enzyme in the lysis buffer. Standard assays were carried out for 1 h at 37 °C. After stopping the reaction by adding chloroform/methanol, Fluorescence was determined at excitation and emission wavelengths of 470 and 530 nm, respectively. The fluorescent peaks were determined by comparing their retention times with standards [11].

### 2.8 SOD activity assay

After being treated with different concentrations of Hst and HTA for 48 h, lysis solution was added to the well. After 5 min, centrifugation of the resulting suspension was done at 14,000*g* at 4 °C for 5 min and then transferred to a clean tube. In the SOD activity assay, superoxide ions generated by xanthine oxidase conversion of xanthine to uric acid and hydrogen peroxide converts NBT to NBT-diformazan, which absorbs light at 560 nm. Optical absorption considered as SOD activity [12].

### 2.9 Statistical analysis

Data were obtained separately, expressed as mean ± S.D. and analyzed with one-way analysis of variance (ANOVA) followed by Tukey׳s to indicate the statistical difference. A P value of 0.05 or less was considered significant. Data were analyzed using SPSS software (version 19.1). IC50 (MTT) and EC50 (caspase assay) values were determined by GraphPad Prism software (version 6.08).
